# Relationship Between Self-Efficacy and Symptoms of Anxiety, Depression, Worry and Social Avoidance in a Normal Sample of Students

**Published:** 2011

**Authors:** Karineh Tahmassian, Niloufar Jalali Moghadam

**Affiliations:** 1 Family Research Institue, Shahid Beheshti University (G.C),; 2 Department of Psychology, Shahid Beheshti University

**Keywords:** Anxiety, Depression, Self-Efficacy, Social Avoidance, Worry

## Abstract

**Objective:** Self-efficacy beliefs determine how people feel, think, motivate themselves and behave. Regarding to similar findings it is supposed that concept of self efficacy has a general role on mental health. The present study focused on examining the relationships between self-efficacy and symptoms of depression, anxiety, worry and social avoidance in a large sample of normal students (n=549).

**Methods:** The sample included of 266 female and 283 male high school students from schools of distinct areas 6, 8 and 9 (Tehran, Iran). The schools were chosen randomly. Participants completed the Self-Efficacy Questionnaire for Children and Social Avoidance & Distress Scale and also the scales measuring trait anxiety, depression, worry and social avoidance. Stepwise regression analyses were used as methods of analysis.

**Results:** Main results distinguished that there is a significant and negative relationship between total self-efficacy, physical self-efficacy and academic self-efficacy and depression. Also significant and negative relationships were found between total self-efficacy, physical self-efficacy and emotional self-efficacy and anxiety. Emotional self-efficacy and physical self-efficacy had significantly a negative relationship to worry. On the other hand, social self-efficacy and physical self-efficacy were significantly and negatively related to social avoidance

**Conclusion:** According to what is discussed the various aspects of mental health is influenced by the sense of self efficacy appraisal. So low self efficacy usually increases some problems such as emotional and social problems which involves in mental health.

## Introduction

Self-efficacy, a construct grounded in social cognitive theory, can be generally defined as personal beliefs in one’s capabilities. Self-efficacy beliefs determine how people feel, think, motivate themselves and behave. Such beliefs produce these diverse effects through four major processes. They include cognitive, motivational, affective and selection processes (1).

A strong sense of efficacy enhances human accomplishment and personal well-being in many ways. Such an efficacious outlook produces personal accomplishments, reduces stress and lowers vulnerability to depression. In contrast, people who doubt their capabilities shy away from difficult tasks which they view as personal threats. They have low aspirations and weak commitment to the goals they choose to pursue. They fall easy victim to stress and depression (2).

On the other hand, adolescence has been described as an important period to acquire social competences required for adult life. It has been suggested that early stressful experiences may be related with the development of psychopathologies such as depression and social anxiety in adulthood (3).

Anxiety disorders predominantly start in childhood and early adolescence, whereas the incidence of mood disorders increase sharply in adolescence and young adulthood (4). Meanwhile, deliberate self-harm behavior--without suicidal intent--is a serious health problem among adolescents. Adolescent and adult self-harmers experience more frequent and more negative emotions, such as anxiety, depression, and aggressiveness, than persons who do not self-harm (5).

Depression, alcohol abuse and suicidality each continue to threaten adolescent populations throughout the world. The comorbidity between these diseases has been found to be up to 73% with consistent positive correlations between adolescent drinking, depression and suicidality (6). Common risk factors for adolescent suicidality include depression and conduct problems (7). Multivariate analyses showed that smoking, bullying, worry, self-harm, and anxiety was associated with self-harm in both genders (8). It is supposed that increasing the sense of self efficacy help managing such unpleasant emotions better and so decreasing the probable harmful outcomes.

Some studies on the contribution of perceived self-efficacy to depression and anxiety have been predominantly confined to adults. Some researchers suggested that the resolution of a self-control conflict has implications for one’s emotional experience and, in addition, people’s emotional experience and mood influence how they resolve a self-control conflict (9). Specifically, researchers have claimed that positive mood impairs self-control because happy (vs. unhappy) people prefer activities that prolong the quest for positive mood & less depressive symptoms (10, 11). 

So far, some research in this area has addressed the role of self-efficacy in early-onset of depression. According to the research results of (2) perceived social and academic inefficacy of children contributed to concurrent and subsequent depression both directly and through their impact on academic achievement, prosocialness, and problem behaviors. Similar outcomes are reported in cross-sectional studies (e.g. 12 and 13). According to study of Ehrenberg self-efficacy has an important relationship with adolescent depression and by regression analysis it was revealed that age-related changes in the dependence of depression scores on general, academic, physical and social self-efficacy status (12). Some have mentioned that Self-efficacy should be regarded as a cognitive precursor or as a component of anxiety and of depression (13).

On the other hand, there are few studies that have examined the relationship between self-efficacy and anxiety which defined anxiety as “a state of anticipatory apprehension over possible deleterious happenings” (14). Individuals experiencing anxiety embody apprehension and avoidant behavior that often interfere with performance in everyday life as well as in academic situations. In social cognitive theory, one’s perceived sense of efficacy plays a key role in the arousal of student anxiety. 

Individuals, therefore, only experience anxiety when they believe themselves to be incapable of managing potentially detrimental events (14). In some studies the relationship between self-efficacy and specific types of anxiety was mentioned (15, 16). 

Consistently, some scientific efforts in this area identified that low levels of self-efficacy are accompanied by high levels of anxiety (17). 

As it is supposed enhancing anxiety lead to an actual sense of worry. So in this study, the relationship between self-efficacy and worry was also investigated. The third pathway of Bandura's agentic model of depression in adolescence and modeling emotional intelligence self-efficacy impacts directly and through worry and academic achievement on adolescents depression. The indirect impact of worry seems to play a more significant role since it is a kind of ruminative and frequent negative thoughts without exercise of control about those thoughts (18).

Emotional inefficacy and worry affect on self regulation and goal orientation in academic functioning (19). According to what these researchers found self-efficacy was highly negatively related to worry.

On the other hand, we have academic efficacy as one of the sub-scales of self-efficacy, so considering it in the study would be interesting. 

In the research of (20) by using hierarchical linear modeling, it was indicated that student reports of the avoidance of help seeking were related to student and classroom characteristics. Avoidance of help seeking was related negatively to students' academic efficacy. 

Obviously, by unbalancing of mental health factors in case of low self efficacy and causing negative outcomes such as depression, anxiety or worry, most part of social behaviors will be affected. 

Data regarding the relationship between self-efficacy and social behavior are limited, and questions remain about how to interpret the relationship of self-efficacy to phobic or avoidance behavior in general (21). 

Thus, it is interesting to study if the correlations between self-efficacy and symptoms of anxiety, depression, worry and social avoidance hold up or not. 

## Materials and Methods

*Participants & Procedure*

The present study is a descriptive research. The main society of research includes girl & boy students of high school & pre-university centers of Tehran. Five-hundred-and-forty-nine adolescents (266 girls and 283 boys; mean AGE=16.5 years, SD= 1.17, range 14-20 years) were recruited from a few high schools of residential areas 6, 8 and 9 in Tehran. The sample groups were selected through clustering sampling.

Participants were asked to complete the set of questionnaires. The teacher and a research assistant were always available to provide assistance if necessary.

*Questionnaires*

*Self-Efficacy Questionnaire for Children*

The SEQ-C contains 24 items that can be divided to three domains of self-efficacy: (1) social self-efficacy (eight items) which has to do with the perceived ability for peer relationships and assertiveness; (2) academic self-efficacy (eight items) which is concerned with the perceived capability to manage one's own learning behavior, to master academic subjects, and to fulfill academic expectations; and (3) emotional self-efficacy (eight items) which pertains to the perceived capability of coping with negative emotions. Each item has to be scored on a five-point scale with 1=not at all and 5=very well. Total self efficacy and subscale scores can be computed by summering across relevant items. 

Murris has gathered some evidence for the validity of the SEQ-C (22). In the sample of 373 adolescents (165 boys and 208 girls) the predicted pattern of correlation between SEQ and scores on active coping (r=0.37, p<0.001) was found. Other studies showed that SEQ has satisfactory reliability and validity (23, 24). 

In present study, the questionnaire was translated to Farsi. The reliability of the SEQ by test-retest of 43 students was 0.89 for total self-efficacy and 0.88 for emotional self-efficacy. Internal consistency (Cronbach's alpha) for emotional self-efficacy was 0.84 and Guttman split-half was 0.82.

*Children Depression Inventory (CDI)*

The CDI (25) is a commonly used self-report measure of depressive symptoms in children and adolescents. The scale has 27 items dealing with sadness, self blame, and loss of appetite, insomnia, interpersonal relationships, and school adjustment. CDI items have to be scored on a three point scale with 0=not true, 1= somewhat true, or 2=very true. A total CDI score can be calculated by summing all scores.

CDI has satisfactory reliability and validity (25). Bandura reported that the alpha reliability coefficient was 0.84 (2). In another research, CDI is reported as a questionnaire that has high levels of consistency, test-retest reliability, and convergent validity (26). They reported that internal consistency was 0.91. In this study, the questionnaire was translated to Farsi. The reliability of CDI by test-retest of 43 students (20 males and 23 females) was 0.83 and Cronbach's alpha was 0.64. The correlation coefficient between CDI and BDI2 was 0.56 (p<0.01) (27).

*Pennstate Worry Questionnaire*

Pennstate Worry Questionnaire (28) is a self report questionnaire measuring frequent negative thoughts. The scale has 14 items and have to be scored on a 4 point scale with 0=not true and 4=very true. Chorpita reported 0.90 for reliability and 0.80 for internal consistency (29). In Iran, Mofrad reported 0.88 for test-retest reliability in a clinical sample and 0.85 in a normal sample (30). 

*Physical Self-efficacy Questionnaire*

Physical Self-efficacy Questionnaire assesses the individual's perception of self physical ability and trust in doing activities and physical skills. This questionnaire has two sub-scales and 22 items. Ten items are related to physical capability sub-scales and assesses the individual's feeling in physical ability and his trust in doing activities that need physical skill. Twelve items are related to the item of physical ability performance. Items have to be scored on six point scale from strongly disagreement=1 to strongly agreement=6. A total score is calculated by summing the scores of two sub-scales. High score in this questionnaire shows high physical self-efficacy. Rikman reported that the alpha reliability coefficient was 0.81 for first sub-scale and 0.84 for second sub-scale and 0.74 for total scale (28). Ehrenberg has reported reliability of 0.80 by test-retest method (p<0.001) (12). Reliability of test, it was calculated 0.81 through test-retest in period of two weeks of 43 students in Iran (27). 

*Spilberger’s State Trait Anxiety Inventory (STAI)*

The questionnaire consists of two scales: the STAI_S that measures present anxiety and the STAI_T that measures the predisposition to anxiety*.*

The goal of this questionnaire is to determine the existence or differences between a subject’s current anxiety as a consequence of a particular situation and the subject’s predisposition to anxiety as a trait of the subject’s personality. In a reliability generalization study for Spielberger’s State-Trait Anxiety Inventory (STAI) a total of 816 research articles utilizing the STAI between 1990 and 2000 were reviewed and classified as having (a) ignored reliability (73%), (b) mentioned reliability or reported reliability coefficients from another source (21%), or (c) computed reliability for the data at hand (6%). Average reliability coefficients were acceptable for both internal consistency and test-retest (31). In Iran, Aghamohammadi reported 0.97 for reliability of the test (32, 33)*.*

*Social Avoidance and Distress Scale*

Social avoidance & distress scale was constructed by Watson & Friend to measure and diagnose of social anxiety (33). This scale concludes two sub-scales: a) social avoidance, & b) social distress. Social avoidance sub-scale assesses isolation and avoidance of social communications in subject. High score is dedicating that the person is highly making himself isolated from social situations and reduce his positive social reinforcements. This scale concludes 26 items. Items have to be scored on a three point scale with the value of 0, 1 and 2. The range of scores is between 0 and 32.

SAD has satisfactory reliability and validity. Watson & Friend reported in their study that retest reliability coefficient was 0.69 (33). Amali also reported reliability coefficient was 0.83 for this test in Iran (34). 

*Statistical Methods*

To examine the relationships between self-efficacy and symptoms of anxiety, depression, worry and social avoidance and predicting the portion of these variables, a series of multiple linear regression by stepwise analyses were carried out with various SEQ-C & PSQ scores being the predictor variables and Spielberg Anxiety Questionnaire, Social Avoidance & distress scale, Pennstate Worry Questionnaire & CDI total scores, as the dependent variables.

## Results

All subscales of self efficacy have a negative relationship with mental health factors such as social anxiety measuring social avoidance, worry, anxiety and depression (Table 1).

**Table 1:** Correlations between subscales of self-efficacy and depression, anxiety, worry and social avoidance

Scales	1	2	3	4	5	6	7	8	9
Social SE	1	0.344†	0.376†	0.473†	0.641†	-0.419†	-0.437†	-0.277†	-0.571†
Academic SE		1	0.321†	0.273†	0.673†	-0.451†	-0.395†	-0.262†	-0.263†
Emotional SE			1	0.348†	0.573†	-0.360†	-0.525†	-0.458†	-0.243†
Physical SE				1	0.423†	-0.457†	-0.471†	-0.353†	-0.387†
Total SE					1	-0.417†	-0.459†	-0.353†	-0.399†
CDI						1	0.775†	0.580†	0.457†
Anxiety							1	0.713†	.432†
Worry								1	.322†
Social Avoidance									1

† Correlation is significant at the 0.01 level (2-tailed).

Noteworthy, the short form of Self-efficacy is embedded whole through the tables for instance (Social SE) refers to Social Self-Efficacy and so on.

Series of regression analyses were carried out with various SEQ-C & PSQ scores being the predictor variables and Anxiety, Social Avoidance Scale, Pennstate Worry Questionnaire & CDI total scores, as the dependent variables.

As can be seen in table 2, total, physical and academic self-efficacy are entered in the analysis and the other variables are excluded (p<0.001). These variables have a significant and negative relationship with CDI.

**Table 2:** Regression analysis to predict depression Score

	B	β	R^2	R	F
Total Self-Efficacy	-0.188	-0.315	0.309	0.556	167.867
Total Self-Efficacy Physical Self-Efficacy	-1.76	1.271	0.359	0.599	104.769
Total Self-Efficacy Physical Self-Efficacy Academic Self-Efficacy	-0.178	1.140	0.367	6.06	72.071

According to results of table 3, total self-efficacy, physical self-efficacy and emotional self-efficacy, have significant and negative relationship with anxiety (p<0.001). Whereas total self-efficacy predict almost 36% of anxiety score, company of total self-efficacy and physical self-efficacy in next step predict 41% of this item, and when emotional self-efficacy add to previous items prediction rate for anxiety convert to almost 43%.

Regression analysis shown in table 4 revealed that, emotional self-efficacy and physical self-efficacy have significant and negative relationship with worry (p<0.001). Other variables are excluded from the analyses. Emotional self-efficacy just predicts 20% of worry whereas combination of emotional self-efficacy and physical self-efficacy predict 25% of this item.

**Table 3:** Regression analysis to predict anxiety score

	B	β	R^2	R	F
Total Self-Efficacy	-0.299	-0.331	0.367	0.606	224.163
Total Self-Efficacy Physical Self-Efficacy	-0.238	-0.244	0.411	0.641	134.289
Total Self-Efficacy Physical Self-Efficacy Emotional Self-Efficacy	-0.420	-0.204	0.429	0.655	96.306

**Table 4:** Regression analysis to predict worry score

	B	β	R^2	R	F
Emotional Self-Efficacy	-0.827	-0.372	0.206	0.454	106.448
Emotional Self-Efficacy Physical Self-Efficacy	-0.254	-0.238	0.256	0.506	70.392

According to table 5, social and physical self-efficacy was significantly and negatively related to self avoidance. Social self-efficacy predict 35% of self avoidance score and in next step by adding physical self-efficacy it increases to 37% of prediction rate

**Table 5:** Regression analysis to predict social avoidance score reases to 37% of prediction rate

	B	β	R^2	R	F
Social Self-Efficacy	-0.552	-0.660	0.351	0.592	22.184
Social Self-Efficacy Physical Self-Efficacy	-0.150	-0.084	0.369	0.607	119.669

The present study examined the relationships between self-efficacy and anxiety, depression, social avoidance and worry in a large sample of normal adolescents. Some support was found for the notion that specific domains of self-efficacy are especially associated with depression, anxiety, worry and social avoidance. That is, total, physical and academic self-efficacy were most strongly connected to depression, total, physical and emotional self-efficacy to anxiety, emotional and physical self-efficacy to worry and social and physical self-efficacy to self avoidance.

According to what was mentioned in introduction social self-efficacy, academic self-efficacy and emotional self-efficacy are involved of negative effect in children and adolescents. Also a low sense of self-efficacy was strongly related to high levels of depression and anxiety.

The results of present study are contingent with those obtained by Ehrenberg MF et al and Comunian AL showing that self-efficacy has an important relationship with depression and anxiety (12, 13). Also Bandura et al, mentioned that social and academic self-efficacy is related to depression and behavioral problems either (2). Bandura indicated that self-efficacy play a unique role of maintenance of negative affective states (14). Murris P suggested that low levels of self-efficacy are accompanied by high levels of anxiety (17).

The results of regression analyses seemed to show that some distinct domains of self-efficacy are related specially to some particular types of pathological symptoms such as worry or social avoidance.

As mentioned before, the third pathway of Bandura's agentic model of depression in adolescence and modeling emotional intelligence self-efficacy impacts directly and through worry and academic achievement on adolescents depression (18). *Malpass* et al also indicated that self-efficacy was highly negatively related to worry (19). On the other hand, physical self-efficacy had significantly a negative relationship to worry in present study. So by increasing physical self-efficacy the sense of worry will decrease. This may be interpreted by considering the important role of physical and somatic efficacy in promoting self esteem and sense of efficacy in young adolescents especially during the years of high school and communicating with peers. As Bandura mentioned a strong sense of efficacy enhances human accomplishment and personal well-being in many ways (1).

There are few studies which have focused on relationship between self-efficacy and social behavior. Ryan et al indicated that avoidance of help seeking was related negatively to students' academic efficacy (20). So if students have a high sense of academic self-efficacy they would have a low level of social avoidance behavior. The outcomes of this research suggested that social avoidance reduce by high sense of social self-efficacy and physical self-efficacy.

As it can be seen, physical self-efficacy is significant variable in relationship with all symptoms (depression, anxiety, social avoidance and worry). Therefore it may play a role in the maintenance of these problems. Since previous studies have not specifically focused on the role of physical self-efficacy that seems very remarkable especially in young people, it would be interesting that further studies examine these different connections between physical self-efficacy and mentioned psychopathological symptoms.

It should be considered that because of the nature of this study it could not follow or find exactly the casual interpretations of the data. So it is not clear yet whether a low sense of self-efficacy results in high levels of psychopathological symptoms or whether high levels of these specific symptoms leads to low sense of self-efficacy. On the other hand since there is some data emphasizing that low self-efficacy should be considered as an antecedent affective disorder like depression (2). Self-efficacy can be best regarded as a cognitive factor that plays a mediating role in the origin of these problems and when people become anxious or depressed, a low sense of self-efficacy will be activated (17).

## Conclusion

Totally, the present results showed that different domains of self-efficacy and symptoms of affective disorders are significantly correlated. But more prospective studies are needed.

## Authors’ contributions

Both authors participated in data collection and conceived anddesigned the evaluation. KT has interpreted the clinical data,performed the statistical analysis and drafted the manuscript. NJM re-analyzed the clinical and statistical data and revised then manuscript. Both authors read and approved the final manuscript.

## Notes

**Declaration of Interest:** None.
